# Impact of interleukin-10, soluble CD25 and interferon-γ on the prognosis and early diagnosis of bacteremic systemic inflammatory response syndrome: a prospective observational study

**DOI:** 10.1186/cc12596

**Published:** 2013-04-05

**Authors:** Giovanni Matera, Rossana Puccio, Aida Giancotti, Angela Quirino, Maria Concetta Pulicari, Emilia Zicca, Santo Caroleo, Attilio Renzulli, Maria Carla Liberto, Alfredo Focà

**Affiliations:** 1 Institute of Microbiology, Department of Health Sciences, "Magna Graecia" University of Catanzaro, Viale Europa, 88100, Catanzaro, Italy; 2 Anesthesiology Unit, Department of Health Sciences, "Magna Graecia" University of Catanzaro, Viale Europa, 88100, Catanzaro, Italy; 3 Cardiac Surgery Unit, Department of Experimental and Clinical Medicine "Magna Graecia" University of Catanzaro, Viale Europa, 88100, Catanzaro, Italy

## Abstract

**Introduction:**

The pathophysiology of sepsis consists of two phases. A first phase characterized by a substantial increase of pro-inflammatory mediators including cytokines and systemic inflammatory markers, and a second phase (immunoparalysis, immunodysregulation) associated with the rise of anti-inflammatory mediators. In this study we prospectively analyzed 52 consecutive patients with diagnosis of systemic inflammatory response syndrome (SIRS) at hospital admission to evaluate prognostic and early diagnostic performance of interleukin-10 (IL-10), soluble CD25 (sCD25) and interferon-γ (IFN-γ) and to confirm the prognostic accuracy of the sequential organ failure assessment (SOFA) score.

**Methods:**

Patients were divided in two groups (group 1, *n *= 28 patients with bacteremic SIRS and group 2, *n *= 24 patients with non-bacteremic SIRS) and then stratified into survivors (*n *= 39) and nonsurvivors (*n *= 13). Serum markers were evaluated on the day of hospital admission (D-1) and on the 7^th ^day of hospital stay (D-7). Concentration of sCD25 was evaluated by a sandwich ELISA kit. Levels of IL-10 and IFN-γ were quantified by a cytokine biochip array by the evidence investigator analyzer. Differences between groups were established by the Mann-Whitney test. Accuracy, sensitivity and specificity of diagnostic markers were evaluated by the receiver-operating characteristic curve analysis. Multivariate analysis was carried out to evaluate whether studied biomarkers are independent predictors of poor outcome in prognosis, and of bacteremic SIRS in diagnosis.

**Results:**

IL-10, sCD25 and SOFA scores of survivors and nonsurvivors were significantly different both at D-1 (*P *= 0.0014; *P *= 0.014 and *P *= 0.0311 respectively) and at D-7 (*P *= 0.0002, *P *= 0.014 and *P *= 0.0012 respectively). Between the above groups IFN-γ level was significantly different only at D-7 (*P *= 0.0013). Moreover IL-10 and sCD25 were significantly higher in bacteremic versus non-bacteremic SIRS patients at D-1 and at D-7 (*P *< 0.05). IFN-γ values showed a significant decrease (*P *< 0.05) in patients of group 1 only at D-7. The diagnostic accuracy of IL-10 and sCD25 was confirmed by the analysis of the AUROCC at D-1 and D-7 respectively. Multivariate analysis revealed that sCD25 and IL-10 are independent predictors of a poor outcome for our patients during the first day of hospital admission.

**Conclusions:**

IL-10 and sCD25 gave a significant contribution to prognostic evaluation and early diagnosis of bacteremic SIRS. SOFA score appeared to be a reliable prognostic tool in this subset of patients.

## Introduction

Sepsis is a severe syndrome with significant morbidity and mortality [1]. The poor knowledge of pathophysiology, the lack of early diagnostic markers and the inability to timely stratify patients with reliable prognostic tools might account for the frequent delay in therapeutic treatment [2]. Although innate immunity and systemic inflammation are generally regarded as a first-line defense against microbial invasion, an overwhelming immune/inflammatory response might contribute to sepsis-related complications [3].

Recently two phases have been identified in the pathophysiology of sepsis: a first phase characterized by a substantial increase of the pro-inflammatory mediators including cytokines, and systemic inflammatory markers, for example, procalcitonin (PCT) and C-reactive protein (CRP), and a second phase (immunoparalysis, immunodysregulation) with the rise of anti-inflammatory mediators [4,5]. The amplitude of the response needs to be fine-tuned in order to achieve effective clearance of pathogens, while limiting the amount of inflammation and avoiding toxicity and collateral tissue damage [6]. Although several candidates have been investigated regarding the anti-inflammatory cascade, the most consistent data concern IL-10 [5]. More recently the soluble form of CD25 (sCD25), a Treg lymphocyte antigen, has been suggested as a marker of the immunosuppressive phase of sepsis [7].

Surgical stress, anesthesia and/or analog sedation can alter and/or compromise the immune response and may disturb the balance of human pro-inflammatory and anti-inflammatory cytokines [4-6]. It has been reported that a significant enhancement of IFN-*γ *and sCD25 release in lipopolysaccharide (LPS)-stimulated whole blood cultures after induction of anesthesia [7]. High IL-10 levels have been associated with a worse outcome after severe sepsis, whereas TNF-α and IL-6 have not [5].

PCT and CRP have already been identified as useful markers of systemic inflammation due to infective (mainly bacterial) agents [8-15]. An effective immune response against bacterial infections requires the development of a T helper (Th)1 response that is associated with the release of IFN-γ [16]. A recent paper [17] suggested a very early role of the adaptive immune system in the pathogenesis of sepsis, hypothesizing that Th1 and Th17 T cells may serve to increase the overall inflammatory response during sepsis. All the above discussed mediators of sepsis pathophysiology might be exploited to gain useful data on the prognosis and early diagnosis of systemic inflammatory response syndrome (SIRS) patients. However, other reports [8,14,15] have questioned the role of PCT, CRP and some cytokines in the clinical management of critically ill patients.

In this scenario the first aim of our study was to investigate the prognostic role of specific mediators (IL-10, sCD25 and IFN-γ) and of the Sequential Organ Failure Assessment (SOFA) score [18] in bacteremic and non-bacteremic SIRS, while the second aim was to assess the early diagnostic role of the above mentioned mediators.

## Materials and methods

### Patients and study design

Fifty-two patients admitted to the University Hospital of Catanzaro (Italy) with diagnosis of SIRS have been sequentially enrolled from May 2008 to April 2009. Twelve healthy volunteers have been enrolled to obtain baseline serum levels of the mediators. We divided patients in two groups: bacteremic SIRS patients (group 1, *n *= 28) with positive blood cultures, and non-bacteremic SIRS patients (group 2, *n *= 24) with persistently negative blood cultures. Most of the included patients (28/52) were submitted to on-pump cardiac surgery. Serum levels have been obtained at D-1 (first day of hospital admission) and D-7 (seventh day of hospital stay). At the end of the observation, patients were stratified into survivors (*n *= 39) and nonsurvivors (*n *= 13). The observational prospective study protocol and the use of volunteers was approved by the Catanzaro University Hospital Ethical Committee. Informed consent was obtained by the patients or by their relatives.

### Inclusion and exclusion criteria

The subjects were enrolled if the diagnosis of SIRS was made. SIRS was defined as two or more of the following: i) hypothermia or fever (temperature < 36°C or > 38.5°C, respectively); ii) tachycardia (> 90 beats/minute); iii) tachypnea (> 20 breaths/minute or PaCO_2 _< 32 mmHg when on mechanical ventilation); iv) leukocytosis (> 12,000 white blood cells (WBCs)/mm^3^), leukopenia (< 4,000 WBCs/mm^3^), or an increase in the number of immature band forms (> 10%) [19]. Subjects under 18 years of age and patients treated with immunosuppressive drugs were excluded from the study. Severity of illness was defined using the SOFA score.

### Laboratory assays

Venous blood samples were obtained from each patient within 6 h from the first day of hospital admission (D-1) and at the seventh day of hospital stay (D-7). Serum was separated, divided in aliquots and immediately frozen (-80 C°) until the time of the assay. Concentrations of sCD25 were evaluated by sandwich ELISA kits, according to the manufacturer's recommendations (Bender MedSystems, Vienna, Austria). Levels of IL-10 and IFN-γ were quantified by a cytokine biochip array on the Evidence Investigator analyser following the manufacturer's instructions (Randox Laboratories Ltd., Crumlin, UK).

Samples for blood cultures were processed with a BacT/Alert 3D system. This system uses blood culture bottles, which include resins that adsorb and neutralize antibiotics contained in the patient's sample. In all blood cultures, growth was usually detected after an incubation of 24 to 36 h. Subcultures on Columbia blood agar, as well as bacteriological stains were carried out on both the direct sampling from the bottle and from the subculture. The microorganisms were identified through the typical Gram stain morphology and the standard clinical microbiology techniques. Isolate identification was confirmed using specific cards processed by a VITEK 2 instrument (bioMerieux, Marcy l'Étoile }France).

### Endpoints

The primary endpoint was to investigate the prognostic role of IL-10, sCD25, IFN-γ and SOFA score in bacteremic and non-bacteremic SIRS, while the secondary endpoint was to assess the early diagnostic value of sCD25, IL-10 and IFN-γ in the same patient population.

### Statistics

Patients were first stratified based on culture results (culture-positive and culture-negative) then subsequently divided into survivors and nonsurvivors. Statistically significant differences between groups were established by the Mann-Whitney test.

The receiver-operating characteristic (ROC) curve was used to evaluate diagnostic accuracy defined by the area under the ROC curve (AUROCC) of the analyzed biomarkers and to determine the sensitivity and specificity at cut-off values selected by Youden index J. The 95% CI for the AUROCC values were estimated using the conservative bootstrap bias-corrected and accelerated method to obtain more accurate intervals [20]. The accuracy of the AUROCC test was defined as: excellent (0.9 to 1.0); good/fair (0.7 to 0.9); poor (0.6 to 0.7); and not useful (< 0.6). Cytokine levels below the limit of detection were assigned a value that was equal to half of the lower limit of detection in the standard curve [21]. Biomarkers evaluated for poor prognosis, as well as for the diagnosis of bacteremic SIRS were analyzed independently using multivariate logistic regression analysis.

The analyses were performed using Graph-Pad 4.0 (Graph-Pad Software Inc., San Diego, CA, USA), SPSS 14.0 software (SPSS, Chicago, IL, USA) and MedCalc 11.1.1.0 Software (BVBA, Mariakerke, Belgium). Results are presented as means ± standard error of the mean (SEM) (unless otherwise stated); *P *< 0.05 was considered significant.

## Results

We enrolled 52 patients (mean age 64 years); 28 patients were culture-positive and 24 were culture-negative (Table 1). In the bacteremic group, the following bacteria were identified: *Staphylococcus spp*. (five isolates), *Pseudomonas spp*. (four isolates), *Candida spp*. (three isolates), *Escherichia coli *(five isolates), *Klebsiella spp*. (three isolates), *Enterococcus faecium *(two isolates), *Shewanella putrefaciens *(one isolate), *Bacteroides capillosus *(one isolate), *Listeria monocytogenes *(one isolate), *Stenotrophomonas maltophilia *(four isolates), *Burkholderia cepacia *(one isolate), *Staphylococcus aureus *(two isolates). A small number of cultures had more than one isolate. Among the bacterial isolates, we found 19 Gram-negative and 11 Gram-positive microorganisms.

**Table 1:** Demographic and clinical features of patients

Demographics	Total *n *= 52	Bacteremic SIRS n *= *28	Non-bacteremic SIRS n *= *24
Mean age, years)	64.07	65.07	64.68
Male/female	39/13	13/15	16/8
Survivors/non-survivors	39/13	16/12	23/1
Diagnosis at hospital/ICU admission			
Cardiac surgery-coronary artery bypass graft	9	5	4
Cardiac surgery of the valvular systems	8	3	5
Combined cardiac surgery	9	5	4
Type I aortic dissection	2	1	1
Cardiogenic pulmonary oedema	5	4	1
Total pneumonectomy	3	2	1
Adult respiratory distress syndrome (ARDS)	2	0	2
Subarachnoid hemorrage	2	2	0
Transient ischemic attack	1	0	1
Lateral Amiotrophic Sclerosis	1	0	1
Meningioma	1	0	1
Meningoencephalitis	1	1	0
Total laringectomy	1	1	0
Total gastrectomy	1	1	0
Post-cardiac arrest recovery	1	0	1
Pneumonia	1	1	0
Polytrauma	1	0	1
Hemoperitoneum	1	1	0
Shock	2	1	1

Results are presented as number of patients. SIRS, systemic inflammatory response syndrome.

### Prognostic roles of markers

**Figure 1:**
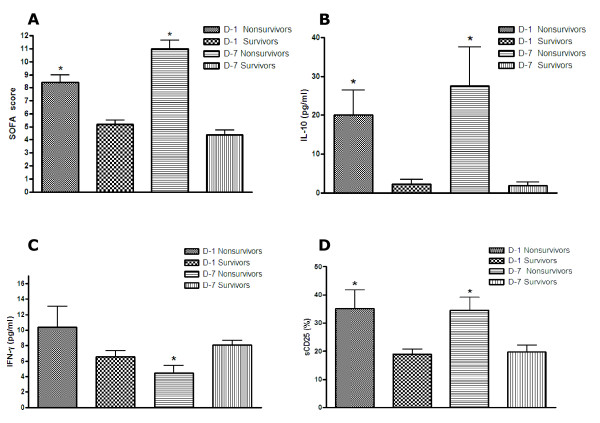
**Sequential Organ Failure Assessment (SOFA) score, IL-10, IFN-γ and sCD25 in non-survivors and survivors SIRS patients**. SOFA score values (**A**), as well as IL-10 (**B**), IFN-γ (**C**) and sCD25 (**D**) serum levels observed in our patients stratified in non-survivors (*n *= 13) and survivors (*n *= 39) at the time of hospital presentation (D-1) and at the end of first week of hospital stay (D-7). Data are means ± standard error of the mean of the observations from the above reported number of patients. *P < 0.05 vs survivors at the same time point (Mann-Whitney test).

As expected, the SOFA scores were significantly higher among nonsurvivors in comparison to survivors at D-1 (*P *= 0.0311) and D-7 (*P *= 0.0012) (Figure 1A).

Serum levels of IL-10 were significantly higher in nonsurvivors at both D-1 (*P *= 0.0014) and D-7 (*P *= 0.0002) (Figure 1B). Similarly, sCD25 exhibited a significant increase among nonsurvivors at both D-1 (*P *= 0.014) and D-7 (*P *= 0.014) (Figure 1D). On the contrary between the above groups IFN-γ level was significantly reduced (Figure 1C) in nonsurvivors at D-7 (*P *= 0.0013). PCT serum levels were also significantly higher in nonsurvivors, but only at D-7 (P = 0.04) (data not shown).

**Figure 2:**
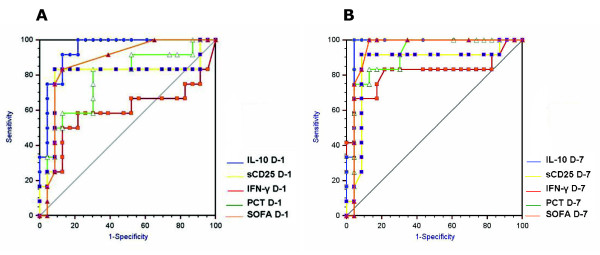
**Receiver-operating characteristic (ROC) analysis of prognostic biomarkers**. ROC curves for IL-10 (blue line), sCD25 (yellow line), IFN-γ (red line), procalcitonin (PCT) (green line) and Sequential Organ Failure Assessment (SOFA) score (orange line) values, observed in our patients stratified in nonsurvivors (*n *= 13) and survivors (*n *= 39) (used as controls for ROC analysis) either at the time of hospital admission (D-1) (**A**) or at the end of first week of hospital stay (D-7) (**B**).

A 25.0% crude mortality was observed in the whole study, with 42.9% in the bacteremic group (Table 1). ROC analysis has been carried out on the studied biomarker data stratified in survivors and non-survivors (Figure 2) to estimate the prognostic value of such biomarkers in terms of the AUROC and the significance AUROC, and the sensitivity, specificity, positive likelihood ratio and negative likelihood ratio. The AUROC of all the biomarkers evaluated was associated with a significant level of *P *(< 0.05) (Table 2).

**Table 2:** Performance of studied variables in predicting mortality

Variable	Parameter	D-1 values (95% CI)	D-7 values (95% CI)
IL-10	AUROCC	0.942 (0.81, 0.99)	0.971 (0.850, 0.99)
	AUROCC significance	*P *= 0.0001	*P *= 0.0001
	Cutoff	3.05	3.40
	Sensitivity	91.67 (61.5, 99.8)	100.00 (73.5, 100.0)
	Specificity	86.96 (66.4, 97.2)	95.65 (78.1, 99.9)
	Positive likelihood ratio	7.03 (2.4, 20.5)	23.00 (3.4, 156.4)
	Negative likelihood ratio	0.096 (0.01, 0.6)	0.00
sCD25	AUROCC	0.793 (0.62, 0.91)	0.861 (0.70, 0.95)
	AUROCC significance	*P *= 0.0047	*P *= 0.0001
	Cutoff	19.9	20.4
	Sensitivity	83.33 (51.6, 97.9)	91.67 (61.5, 99.8)
	Specificity	91.30 (72.0, 98.9)	91.30 (72.0, 98.9)
	Positive likelihood ratio	9.58 (2.5, 36.9)	10.54 (2.8, 40.1)
	Negative likelihood ratio	0.18 (0.05-0.7)	0.091 (0.01-0.6)
IFN-γ	AUROCC	0.594 (0.415, 0.756)	0.815 (0.648, 0.926)
	AUROCC significance	*P *= 0.4447	*P *= 0.0011
	Cut-off	9.00	5.79
	Sensitivity	50.00 (21.1, 78.9)	66.67 (34.9, 90.1)
	Specificity	86.96 (66.4, 97.2)	95.65 (78.1, 99.9)
	Positive likelihood ratio	3.83 (1.2, 12.7)	15.33 (2.2, 108.7)
	Negative likelihood ratio	0.58 (0.3, 1.0)	0.35 (0.2, 0.8)
PCT	AUROCC	0.768 (0.595, 0.893)	0.904 (0.756, 0.977)
	AUROCC significance	*P *= 0.0027	*P *= 0.0001
	Cutoff	0.35	0.87
	Sensitivity	83.33 (51.6, 97.9)	75.00(42.8, 94.5)
	Specificity	69.57 (47.1, 86.8)	95.65 (78.1, 99.9)
	Positive likelihood ratio	2.74 (1.4, 5.3)	17.25 (2.5, 120.6)
	Negative likelihood ratio	0.24 (0.07, 0.9)	0.26 (0.10, 0.7)
SOFA	AUROCC	0.870 (0.713, 0.959)	0.944 (0.810, 0.993)
	AUROCC significance	*P *= 0.0001	*P *= 0.0001
	Cutoff	6	7
	Sensitivity	83.33(51.6, 97.9)	100.00 (73.5, 100.0)
	Specificity	86.96 (66.4, 97.2)	86.96 (66.4, 97.2)
	Positive likelihood ratio	6.39 (2.2, 18.9)	7.67 (2.7, 22.0)
	Negative likelihood ratio	0.19 (0.05, 0.7)	0.00

Prognostic accuracy of IL-10, sCD25, IFN-γ, procalcitonin (PCT) and Sequential Organ Failure Assessment (SOFA) score evaluated by receiver-operating characteristic (ROC) analysis carried out on samples obtained at the time of hospital presentation (D-1) and at the end of first week of the hospital stay (D-7), to estimate the pro- and anti-inflammatory mediators as prognostic markers of poor outcome in critically ill patients. AUROCC, area under the ROC curv

In the multivariate analysis of the logistic regression model, using the SOFA score for correction of disease severity, sCD25 and IL-10 were the variables with statistically significant relative risks at time D-1, and therefore could be considered to be independent predictors of a poor outcome for our patients during the first day of hospital admission (Table 3).

**Table 3:** Multivariate analysis of the factors associated with prognosis of bacteremic patients with systemic inflammatory response syndrome (SIRS)

Factor		Odds ratio	95% (CI)	*P*-value
IL-10	D-1	1.86	0.98, 3.52	< 0.05
	D-7	2.06	0.14, 6.54	> 0.05
sCD25	D-1	1.12	1.01, 1.25	< 0.05
	D-7	0.97	0.87, 1.09	> 0.05
IFN-γ	D-1	1.03	0.86, 1.23	> 0.05
	D-7	0.54	0.19, 1.51	> 0.05
PCT	D-1	6.53	0.06, 68.4	> 0.05
	D-7	39.71	0.05, 315	> 0.05

PCT: procalcitonin.

### Diagnostic role of markers

**Figure 3:**
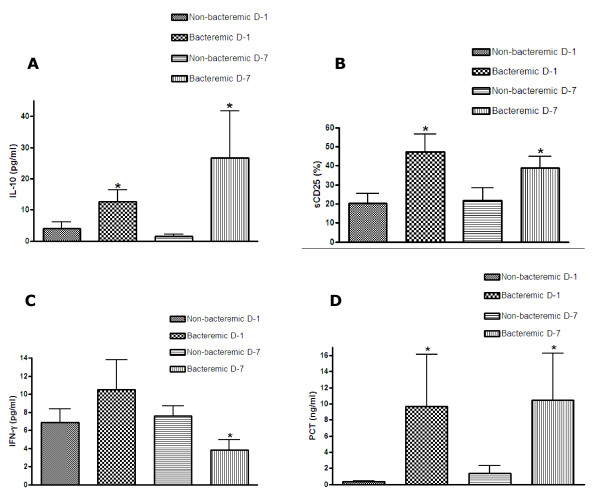
**IL-10, sCD25, IFN-γ and procalcitonin (PCT) levels in bacteremic and non-bacteremic patients with systemic inflammatory response syndrome (SIRS)**. Levels of IL-10 (**A**), sCD25 (**B**), IFN-γ (**C**), and PCT (**D**) in serum samples of the bacteremic (culture-positive) and non-bacteremic (culture-negative) SIRS patients at the time of hospital admission (D-1) and at the end of first week of the hospital stay (D-7). Data are presented as means ± standard error of the mean of data from either 28 bacteremic or 24 non-bacteremic patients. *P < 0.05 vs non-bacteremic patients at the same time point (Mann-Whitney test).

The trend of IL-10 levels showed a significant increase (*P *< 0.05) in the group of bacteremic patients at D-1 and D-7 (Figure 3A). AUROCC values at both of the time intervals were fairly high, as well as their sensitivity and specificity (Table 4).

**Table 4:** Performance of the studied variables in the diagnosis of bacteremic systemic inflammatory response syndrome (SIRS)

Variable	Parameter	D-1 values (95% CI)	D-7 values (95% CI)
IL-10	AUROCC	0.767 (0.601, 0.888)	0.753 (0.579, 0.883)
	AUROCC significance	*P *= 0.0021	*P *= 0.0016
	Cutoff	3.05	0.59
	Sensitivity	78.26 (56.3, 92.5)	66.67 (43.0, 85.4)
	Specificity	80.0 (51.9, 95.7)	71.43 (41.9, 91.6)
	Positive likelihood ratio	3.91 (2.8, 5.5)	2.33 (1.5, 3.7)
	Negative likelihood ratio	0.27 (0.08, 1.0)	0.47 (0.2, 1.3)
sCD25	AUROCC	0.812 (0.544, 0.960)	0.785 (0.523, 0.943)
	AUROCC significance	*P *= 0.0095	*P *= 0.0201
	Cutoff	19.9	22.1
	Sensitivity	87.5 (47.3, 99.7)	87.5 (47.3, 99.7)
	Specificity	75.0 (34.9, 96.8)	77.78 (40.0, 97.2)
	Positive likelihood ratio	3.5 (2.2, 5.6)	3.94 (2.5, 6.1)
	Negative likelihood ratio	0.17 (0.02, 1.5)	0.16 (0.02, 1.5)
IFN-γ	AUROCC	0.486 (0.266, 0.711)	0.745 (0.504, 0.910)
	AUROCC significance	*P *= 0.9204	*P *= 0.0342
	Cut-off	9.0	1.8
	Sensitivity	45.45 (16.7, 76.6)	60.0 (26.2, 87.8)
	Specificity	70.0 (34.8, 93.3)	90.0 (55.5, 99.7)
	Positive likelihood ratio	1.52 (0.7, 3.3)	6.0 (3.5, 10.4)
	Negative likelihood ratio	0.78(0.3-2.3)	0.44 (0.06-3.3)
PCT	AUROCC	0.800 (0.641, 0.911)	0.781(0.612, 0.901)
	AUROCC significance	*P *= 0.0001	*P *= 0.0011
	Cutoff	0.24	0.32
	Sensitivity	91.3 (72.0, 98.9)	95.24 (76.2, 99.9)
	Specificity	62.5 (35.4, 84.8)	60.0 (32.3, 83.7)
	Positive likelihood ratio	2.43 (1.6, 3.6)	2.38 (1.6, 3.6)
	Negative likelihood ratio	0.14 (0.03, 0.6)	0.079 (0.01, 0.6)
CRP	AUROCC	0.56 (0.392, 0.718)	0.58 (0.407, 740)
	AUROCC significance	*P *= 0.5276	*P *= 0.4055
	Cutoff	137	125
	Sensitivity	39.13 (19.7, 61.5)	50.0 (28.2, 71.8)
	Specificity	87.5 (61.7, 98.4)	80.0 (51.9, 95.7
	Positive likelihood ratio	3.13 (1.8, 5.4)	2.50 (1.5, 4.1)
	Negative likelihood ratio	0.70 (0.2, 2.6)	0.62 (0.2, 1.9)

Diagnostic accuracy of IL-10, sCD25, IFN-γ, procalcitonin (PCT) and C-reactive protein (CRP) evaluated by receiver-operating characteristics (ROC) analysis carried out on samples obtained at the time of hospital presentation (D-1) and at the end of the first week of the hospital stay (D-7), to estimate the pro- and anti-inflammatory mediators as diagnostic markers of bacteremic SIRS in critically ill patients. AUROCC, area under the ROC curve.

Similarly sCD25 serum values were significantly higher (*P *< 0.05) in the bacteremic group at both D-1 and D-7 (Figure 3B). The values of AUROCC showed high sensitivity and specificity at D-1 and D-7 with significant accuracy for sCD25 (Table 4). IFN-γ levels were significantly lower in bacteremic patients vs non-bacteremic patients at D-7 only (Figure 3C), when the accuracy of the AUROCC of the biomarker was also significant (*P *< 0.05). Serum levels of PCT were significantly higher in bacteremic patients at D-1 and D-7 compared to non-infectious SIRS (*P *< 0.05) (Figure 3D).

**Figure 4:**
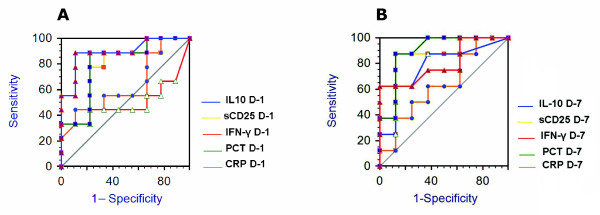
**Receiver-operating characteristic (ROC) analysis of diagnostic biomarkers**. ROC curves for values of IL-10 (blue line), sCD25 (yellow line), IFN-γ (red line), procalcitonin (PCT) (green line) and C-reactive protein (CRP) (orange line), observed in our patients stratified as culture-positive or culture-negative (used as controls for ROC analysis), either at the time of hospital admission (D-1) (**A**) or at the end of first week of the hospital stay (D-7) (**B**).

The mean values of serum levels of IFN-γ and IL-10 in healthy volunteers were 1.57 ± 0.52 pg/ml and 0.56 ± 0.16 pg/ml respectively, while sCD25 concentration was not measurable. A statistically significant difference (*P *< 0.05) was observed between levels of the measurable markers between the controls and bacteremic patients. The ROC curves of diagnostic biomarkers evaluated at D-1 and at D-7 are showed in panel A and B respectively of Figure 4. The multivariate analysis of diagnostic biomarkers is reported in Table 5; none of the investigated biomarkers appeared to be independent predictors of bacteremic SIRS in our patients.

**Table 5:** Multivariate analysis of the factors associated with diagnosis of bacteremic patients with systemic inflammatory response syndrome (SIRS)

Factor		Odds ratio	95% (CI)	*P*-value
IL-10	D-1	1.01	0.87, 1.16	> 0.05
	D-7	0.97	0.70, 1.34	> 0.05
sCD25	D-1	1.11	0.97, 1.25	> 0.05
	D-7	1.08	0.97, 1.22	> 0.05
IFN-γ	D-1	1.08	0.88, 1.32	> 0.05
	D-7	0.40	0.12, 1.33	> 0.05
PCT	D-1	3.86	0.16, 91.93	> 0.05
	D-7	1.96	0.51, 7.61	> 0.05
CRP	D-1	1.02	0.97, 1.05	> 0.05
	D-7	0.95	0.89, 1.02	> 0.05

PCT: procalcitonin; CRP: C-reactive protein.

**Figure 5:**
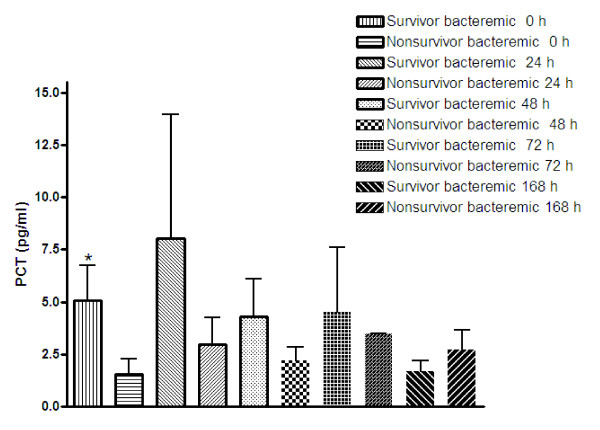
**Serial measurements of procalcitonin (PCT) levels in the serum samples from bacteremic survivors and bacteremic nonsurvivors**. PCT levels were evaluated at 0, 24, 48, 72, and 168 h after admission. *P < 0.05 vs non-surviving bacteremic patients at the same time point (Mann-Whitney test).

Serum levels of PCT were analyzed and levels of the marker were observed at several time points (0, 24, 48, 72, and 168 hours after admission) in bacteremic survivors and nonsurvivors (Figure 5). Only during the hospital admission day (0 h) was there a significant (*P *= 0.0075) difference between the above subset of bacteremic patients. Indeed bacteremic survivors exhibited a higher level of PCT until 72 h after admission, although PCT was only significant on hospital admission day.

## Discussion

Taken together our data suggest that IL-10 and sCD25 may be considered as relevant markers of prognosis, in addition to the SOFA score in this specific clinical context. Moreover IL-10 and sCD25 might have a major role in the early diagnosis of bacteremic SIRS. Until now inconclusive attempts have been made to identify an optimal marker of sepsis [13,14]. The ideal parameter should be sensitive enough to perceive the presence of pathogens with a minimal host response and at the same time specific enough to distinguish between infectious and non-infectious systemic inflammatory response. Moreover it should be easily and rapidly available and finally it should be reliable from a prognostic point of view [22].

The availability of a reliable marker for early diagnosis is still an unsolved problem. Some reports have questioned the role of PCT, CRP and some cytokines for the diagnosis and the prognosis of critically ill patients [14,15]. A more recent article [23] showed a moderate diagnostic performance of PCT, with mean values of 71% (95% CI 67 to 76%) for both sensitivity and specificity, and AUROCC of 0.78 (95% CI 0.73 to 0.83). These values are similar to our findings (0.80 for D-1 and 0.78 for D-7) but different from AUROCC values (0.92) shown in other reports [24]. Moreover, our findings of bacteremic survivors exhibiting a higher level of PCT in comparison to bacteremic nonsurvivors until 72 h after admission (although only significant was on hospital admission day), probably warrant further assessment.

Our results showed a statistically non-significant increase of IFN-γ among bacteremic SIRS patients at D-1 followed by a statistically significant decrease of the same biomarker in this group of patients at D-7. In accordance with our data, other authors showed a non-significant increase of both IFN- γ and its mRNA in septic patients [25]. However, other data about the early increase of IFN- γ during sepsis and human experimental endotoxemia have been previously collected [26]. At the same time, the significance of the IFN- γ decrease in late sepsis in both human [27] and animal settings [28] has been confirmed. We could speculate that the decrease of IFN- γ values observed in this study may indicate the beginning of compensatory antiinflammatory response syndrome (CARS) [29] during the late stage of sepsis.

Lymphocyte dysfunction has been related to profound immune depression, eventually leading to septic shock and poor outcome [30]. Lymphocyte subpopulations differentially undergo apoptosis during sepsis with a high resistance within the CD25^+ ^subset [5]: in this context it is therefore likely that these cells may give their suppressive contribution through the release of sCD25 and IL-10 [31]. Our findings on IL-10 demonstrate the prognostic and diagnostic value of this cytokine in a specific clinical scenario. Due to its anti-inflammatory and immunosuppressive activity, IL-10 may initially control the exaggerated pro-inflammatory wave of early mediators in sepsis; however persistent high levels of IL-10 can cause immunoparalysis and eventually lead to poor outcome in septic shock [5]. A quite unexpected finding of our study was the early increase of IL-10 and sCD25, which should be associated with the late/immunosuppressive stage of sepsis according to current literature [4,5]. Very recently Kasten *et al. *[17] underlined a very early role of the adaptive immune system in the pathogenesis of sepsis. Therefore, IL-10 and sCD25 should be considered not only as late but also as early mediators of bacteremic SIRS and sepsis. Enhanced concentrations of IL-10 associated with a decrease of the IFN- γ might account for the delay in pathogen eradication during the late stage of sepsis (immunoparalysis) [5]. An increase of soluble CD25 in the plasma of bacteremic compared with non-bacteremic SIRS patients has been recently reported [7]. In accordance with these reports our study has highlighted the prognostic and early diagnostic value of IL-10 and sCD25: we believe that in the near future these markers could become valid tools for the management of patients with bacteremic SIRS and sepsis.

### Limitations

Our study has several limitations. First of all this is an observational prospective investigation with quite a small sample size. The final results of microbiological cultures are not necessarily representative of the rest of the country or of other nations. The small sample size is mainly due to missing data from many of our bacteremic and non-bacteremic SIRS patients, for whom informed consensus was not obtained, or to lack of the required sampling or of timely sampling carried out by nurses. These patients were not included in the study. Also the difficulties in finding SIRS patients with comparable age, sex and co-morbidities often made matching difficult. Further limitation of our study is the small number of the blood samples evaluated for the biomarkers; unfortunately no data are available in the middle of the follow up period.

## Conclusions

sCD25 and IL-10 have been identified as reliable prognostic tools in this context. Moreover, their diagnostic accuracy could give a significant contribution to the early identification of the patients with bacteremic SIRS. The good prognostic performance of the SOFA score has been confirmed.

## Key messages

• IL-10 and sCD25 have emerged as valid prognostic and early diagnostic tools in the clinical course of bacteremic SIRS.

• In particular sCD25 seems to be helpful for the clinicians to monitor the patients admitted to the hospital with signs of SIRS, and to address the early therapeutic approach.

## Abbreviations

AUROCC: area under the receiver-operating characteristic curve; CARS: compensatory antiinflammatory response syndrome; ELISA: enzyme-linked immunosorbent assay; IFN-γ: gamma interferon; IL: interleukin; LPS: lipopolysaccharide; PCT: procalcitonin; ROC: receiver-operating characteristic; sCD25: soluble CD25; SOFA:, Sequential Organ Failure Assessment; SEM: standard error of the mean; SIRS: systemic inflammatory response syndrome; TNF-α: tumor necrosis factor α.

## Competing interests

The authors declare that they have no competing interests.

## Authors' contributions

GM conceived the study, drafted the manuscript and participated in its design. RP carried out bacteriological cultures and identification of microorganisms. AG carried out CRP and PCT assays and supervised cytokine assays. AQ participated in bacteriological cultures and helped to draft the manuscript. MCP contributed to the cytokine studies, collected the data and performed statistical analysis. EZ carried out cytokine assays and helped to perform CRP and PCT studies. SC and AR carried out the clinical studies and contributed to statistical analysis. MCL participated in the design and coordination of the study, and contributed in the drafting and editing of the manuscript. AF conceived the study and participated in its design and coordination. All authors read and approved the final manuscript.

## References

[B1] Wang EH Devereaux RS Yealy DM Safford MM Howard G National variation in United States sepsis mortality: a descriptive study Int J Health Geogr 2010 17 9 10.1186/1476-072X-9-9 20156361 PMC2831852

[B2] Dombrovskiy VY Martin AA Sunderram J Paz HL Rapid increase in hospitalization and mortality rates for severe sepsis in the United States: a trend analysis from 1993 to 2003 Crit Care Med 2007 17 1244 1250 10.1097/01.CCM.0000261890.41311.E9 17414736

[B3] Remick DG Pathophysiology of sepsis Am J Pathol 2007 17 1435 1444 10.2353/ajpath.2007.060872 17456750 PMC1854939

[B4] Annane D Bellissant E Cavaillon JM Septic shock Lancet 2005 17 63 78 10.1016/S0140-6736(04)17667-8 15639681

[B5] Monneret G Venet F Pachot A Lepape A Monitoring immune dysfunctions in the septic patient: a new skin for the old ceremony Mol Med 2008 17 64 78 18026569 10.2119/2007-00102.Monneret PMC2078557

[B6] Cohen J The immunopathogenesis of sepsis Nature 2002 17 885 891 10.1038/nature01326 12490963

[B7] Saito K Wagatsuma T Toyama H Ejima Y Hoshi K Shibusawa M Kato M Kurosawa S Sepsis is Characterized by the increases in percentages of circulating CD4+ CD25+ regulatory T cells and plasma levels of soluble CD25 Tohoku J Exp Med 2008 17 61 68 10.1620/tjem.216.61 18719339

[B8] Becker KL Snider R Nylen ES Procalcitonin assay in systemic inflammation, infection, and sepsis: Clinical utility and limitations Crit Care Med 2008 17 941 952 10.1097/CCM.0B013E318165BABB 18431284

[B9] Maruna P Nedẽlnỉkovă K Gűrlich R Physiology and Genetics of procalcitonin Physiol Res 2000 17 S57 S61 10984072

[B10] Assicot M Gendrel D Carsin H Raymond J Guilbaud J Bohuon C High serum procalcitonin concentrations in patients with sepsis and infection Lancet 1993 17 515 518 10.1016/0140-6736(93)90277-N 8094770 PMC7141580

[B11] Castelli GP Pognani C Meisner M Stuani A Bellomi D Sgarbi L Procalcitonin and C-reactive protein during systemic inflammatory response syndrome, sepsis, and organ dysfunction Crit Care 2004 17 R234 R242 10.1186/cc2877 15312223 PMC522844

[B12] Povoa P C-reactive protein a valuable marker of sepsis Intensive Care Med 2002 17 235 243 10.1007/s00134-002-1209-6 11904651

[B13] Spapen HD Hachimi-Idrissi S Corne L Huyghens LP Diagnostic markers of sepsis in the emergency department Acta Clin Belg 2006 17 138 142 16881563 10.1179/acb.2006.022

[B14] Pierrakos C Vincent JL Sepsis biomarkers: a review Crit Care 2010 17 R15 10.1186/cc8872 20144219 PMC2875530

[B15] De Bard AL Vautrin C Pariset C Bienvenu J Monneret G High serum procalcitonin levels do not predict bacteremia in adult patiens with acute fever Clin Infect Dis 2003 17 825 826 10.1086/368095 12627373

[B16] Flohé SB Agrawal H Flohé S Rani M Bangen JM Schade FU Diversity of Interferon γ and Granulocyte-Macrophage Colony-Stimulating Factor in Restoring Immune Dysfunction of Dendritic Cells and Macrophages During Polymicrobial Sepsis Mol Med 2008 17 247 256 18297128 10.2119/2007-00120.Flohe PMC2249752

[B17] Kasten KR Tschöp J Goetzman HS England LG Dattilo JR Cave CM Seitz AP Hildeman DA Caldwell CC T-cell activation differentially mediates the host response to sepsis Shock 2010 17 377 383 20610943 10.1097/SHK.0b013e3181dc0845

[B18] Vincent JL Moreno R Takala J Willatts S De Mendonça A Bruining H Reinhart CK Suter PM Thijs LG The SOFA (Sepsis-related Organ Failure Assessment) score to describe organ dysfunction/failure Intensive Care Med 1996 17 707 710 10.1007/BF01709751 8844239

[B19] Rangel-Frausto MS Pittet D Hwang T Woolson RF Wenzel RP The dynamic of disease progression in sepsis: Markov modeling describing the natural history and the likely impact of anti-septic agents Clin Infect Dis 1998 17 185 190 10.1086/514630 9675475

[B20] Lasko TA Bhagwat JG Zou KH Ohno-Machado L The use of receiver operating characteristic curves in biomedical informatics J Biomed Inform 2005 17 404 415 10.1016/j.jbi.2005.02.008 16198999

[B21] Osuchowski MF Welch K Yang H Siddiqui J Remick DG Chronic sepsis mortality characterized by an individualized inflammatory response J Immunol 2007 17 623 630 17579084 10.4049/jimmunol.179.1.623 PMC4429887

[B22] Marshall JC Sepsis: rethinking the approach to clinical research J Leukoc Biol 2008 17 471 482 18171697 10.1189/jlb.0607380

[B23] Tang BMP Eslick GD Craig JC McLean AS Accuracy of procalcitonin for sepsis diagnosis in critically ill patients: systematic review and meta-analysis Lancet Infect Dis 2007 17 210 217 10.1016/S1473-3099(07)70052-X 17317602

[B24] Harbarth S Holeckova K Pittet D Ricou B Grau GE Vadas L Pugin J Geneva Sepsis Network Diagnostic value of procalcitonin, interleukin-6, and interleukin-8 in critically ill patients admitted with suspected sepsis Am J Respir Crit Car Med 2001 17 396 402 10.1164/ajrccm.164.3.2009052 11500339

[B25] Abe R Hirasawa H Oda S Sadahiro T Nakamura M Watanabe E Nakada TA Hatano M Tokuhisa T Up-regulation of interleukin-10 mRNA expression in peripheral leukocytes predicts poor outcome and diminished human leukocyte antigen-DR expression on monocytes in septic patients J Surg Res 2008 17 1 8 10.1016/j.jss.2007.07.009 17720196

[B26] Dorresteijn MJ Pickkers P Netea MG Van der Hoeven JG IFN-gamma is not induced through increased plasma concentrations of interleukin-12/interleukin-18 during human endotoxemia Eur Cytokine Netw 2005 17 191 193 16266858

[B27] O'Dwyer MJ Mankan AK Stordeur P O' Connell B Duggan E White M Kelleher DP McManus R Ryan T The occurrence of severe sepsis and septic shock are related to distinct patterns of cytokine gene expression Shock 2006 17 544 550 10.1097/01.shk.0000235091.38174.8d 17117127

[B28] Murphy TJ Paterson HM Mannick JA Lederer JA Injury, sepsis, and the regulation of Toll-like receptor responses J Leukoc Biol 2004 17 400 407 14557385 10.1189/jlb.0503233

[B29] Ward NS Casserly B Ayala A The compensatory anti-inflammatory response syndrome (CARS) in critically ill patients Clin Chest Med 2008 17 617 625 10.1016/j.ccm.2008.06.010 18954697 PMC2786900

[B30] Le Tulzo Y Pangault C Gacouin A Guilloux V Tribut O Amiot L Tattevin P Thomas R Fauchet R Drénou B Early Circulating lymphocyte apoptosis in human septic shock is associated with poor outcome Shock 2002 17 487 494 10.1097/00024382-200212000-00001 12462554

[B31] Vignali DAA Collison LW Workman CJ How regulatory T cells work Nat Rev Immunol 2008 17 523 532 10.1038/nri2343 18566595 PMC2665249

